# Medical Thermometers

**Published:** 1800-04

**Authors:** 


### MEDICAL THERMOMETERS.

Dr. Currie, in his excellent work on Fever, having evinced the great benefit often derived from the affufion of cold water, Practitioners in the Army and Navy, as well as the Phyficians to public Inftitutions, became defirous of availing themfelves of the ufe of a remedy fo cheap, pleafant, and efficacious. For this purpofe, it was neceffary to afcertain the heat of the body with a degree of precifion, for which the hand of the practitioner can feldom be relied on: thermometers were therefore recommended; and we have at length obtained a fpecimen that appears perfectly fatisfactory. The fcale is attached to the tube, and the whole inftrument is contained in a cylindrical cafe about five inches long, and a quarter of an inch in diameter; therefore fufficiently portable.

As this inftrument is defigned for the fole purpofe of afcertaining the heat of the human body, its range is very limited, in order to obtain the requifite fenfibility: it extends from about 80 degrees to 112; and is fo fenfible, that it will indicate the heat applied to it in lefs than ten feconds; and the fcale may be read to a quarter of a degree. It will be fcarcely neceffary to caution our readers againft immerfing it in fluids of a temperature higher than 112°, as it might endanger the inftrument.

Gentlemen in the country may be fupplied with fuch Thermometers as above defcribed, or with thofe of more extenfive fcales, if defired, by Allen and Howard, chemifts, in Plough-court, Lombard-ftreet, at about 18s. each.

